# Characterization and Crystal Nucleation Kinetics of a New Metastable Polymorph of Piracetam in Alcoholic Solvents

**DOI:** 10.1021/acs.cgd.1c01421

**Published:** 2022-04-22

**Authors:** Shubhangi Kakkar, Lai Zeng, Michael Svärd, Åke C. Rasmuson

**Affiliations:** † SSPC, Bernal Institute, Department of Chemical Sciences, University of Limerick, Limerick V94 T9PX, Ireland; ‡ Department of Chemical Engineering, KTH Royal Institute of Technology, Stockholm SE-100 44, Sweden

## Abstract

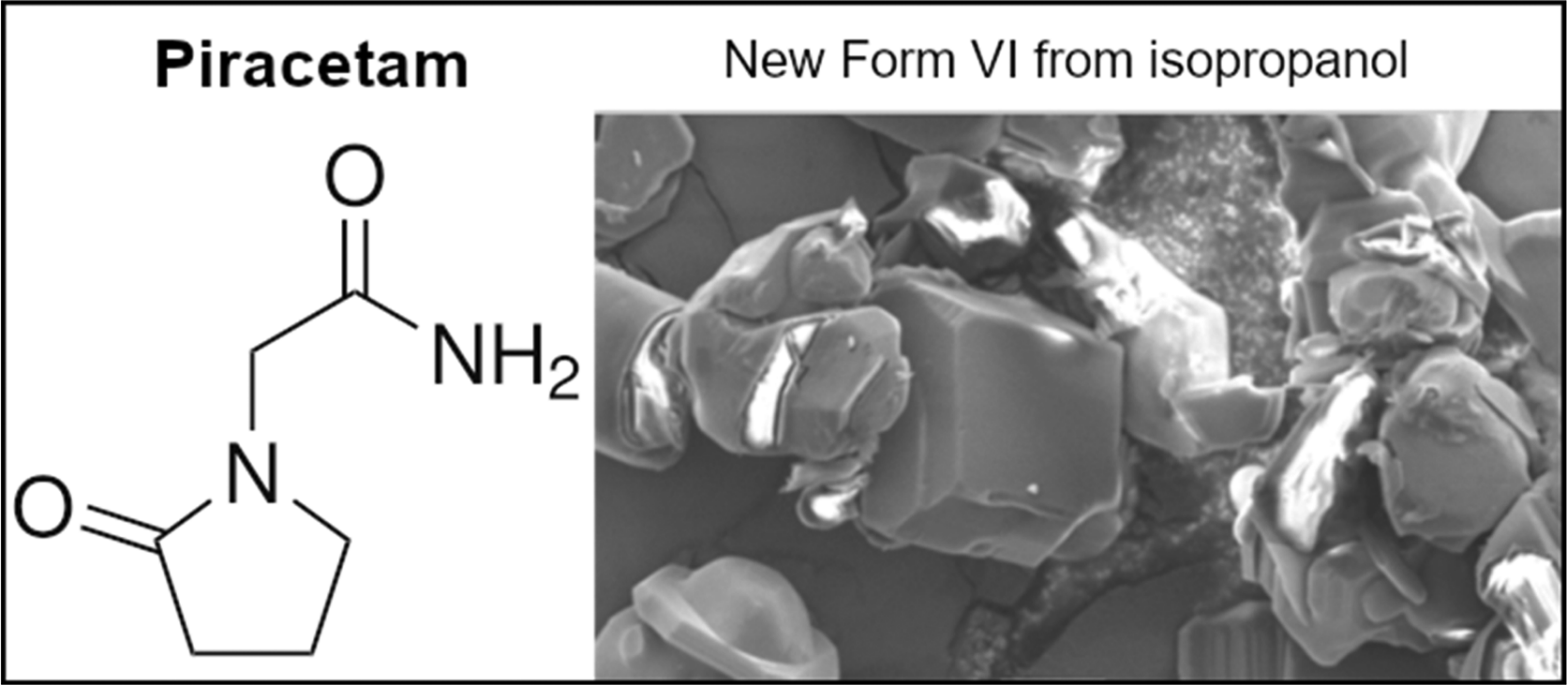

A new polymorph of the drug active pharmaceutical ingredient piracetam (Form VI) has been discovered and characterized by X-ray powder diffraction (PXRD), solid-state Raman, attenuated total reflectance infrared spectroscopy, and differential scanning calorimetry. The PXRD diffractogram of Form VI shows a distinct peak at 24.2° (2θ) that distinguishes it from the previously known polymorphs and solvates. Form VI is metastable with respect to the previously known polymorphs Form II and Form III; in ethanol solution at 288 K, Form VI transforms into Form II within 15 min, while in isopropanol solution Form VI is kinetically stable for at least 6 h. A total of 1200 crystal nucleation induction time experiments of piracetam in ethanol and isopropanol solutions have been conducted, in sets of 40–80 repeat experiments carried out at different temperatures and solute concentrations. Each solution nucleated as a single polymorph, and each set of repeat experiments resulted in different proportions of Form II, Form III, and Form VI, with Form VI dominating at low nucleation temperatures and Form II at higher nucleation temperatures. The induction time data for Form VI at 288 K have been evaluated within the framework of the classical nucleation theory. At equal driving force, nucleation of Form VI is less obstructed in ethanol than in isopropanol, as captured by a lower interfacial energy and higher pre-exponential factor in ethanol. The proportion of Form VI obtained at a comparable driving force increases in the order ethanol < isopropanol.

## Abstract 2

A new metastable polymorph of the drug active pharmaceutical ingredient piracetam (Form VI) has been discovered and characterized. A total of 1200 induction time experiments at different temperatures, concentrations, and supersaturations are reported. Nucleation of the new Form VI dominates under most conditions, except at high temperature. At equal driving force, nucleation of Form VI is less obstructed in ethanol than in isopropanol.

## 1 Introduction

Crystallization from solution is a common unit operation in the pharmaceutical industry. In crystallization processes, primary nucleation is of crucial importance for the resulting product properties. The fundamental mechanisms underlying nucleation are not well understood; thus, nucleation tends to be unpredictable. Controlling nucleation is necessary to control particle size, size distribution, and polymorphic form, which is further affected by many factors, such as temperature and supersaturation.^1,2^ Different solid forms can lead to variations in product performance, such as solubility, dissolution rate, or tablet hardness. Polymorphism can lead to dramatic effects in biological activity between two forms of the same drug. In 1999, Griesser and Burger reported that out of 953 drug molecules tested, more than 59% are known to exist in more than one crystal form.^3^ During the late 20th and early 21st centuries, many drugs were recalled^4,5^ in the United States and Europe because of the unexpected appearance of a new polymorph, e.g., Ritonavir (Norvir) and Rotigotine (Neupro).

In polymorphic systems, the overall effect of the solvent on nucleation can be very important for the outcome.^6−12^ Davey et al. explored the link between supramolecular structuring in solution and the polymorph nucleating from different solvents, for the compound 2,6-dihydoxybenzoic acid.^7^ Gracin et al.^13^ in a study of *p*-aminobenzoic acid, an enantiotropic polymorphic system, showed that the influence of the solvent on nucleation could be explained by analyzing the crystal structure and the possible solute–solvent interactions in the solution. Chiarella et al.^14^ in another nucleation study of inosine with a combination of computational and experimental tools explored the relationship between the solution-phase inosine species and the structural synthons present in its crystal structures. Overall, the mechanisms behind the influence of the solvent are insufficiently understood, and the polymorphic outcome cannot be predicted. Thus, more studies are required to understand these mechanisms, which can differ greatly between different systems.

**Figure 1:**
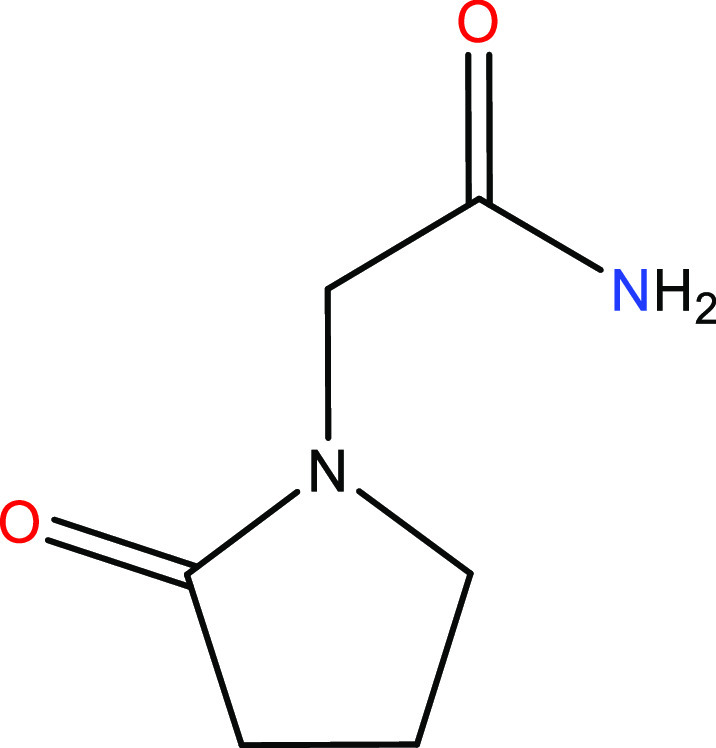
Two-dimensional structure of piracetam (2-(2-oxopyrrolidin-1-yl)-acetamide).

Piracetam (PCM, 2-(2-oxopyrrolidin-1-yl)-acetamide), shown in Figure 1, is a nootropic agent used for memory enhancement in humans. Piracetam is approved in many European countries for myoclonus and aging-related conditions like Alzheimer’s disease. It is an effective drug for memory dysfunction, alcoholism, Raynaud’s phenomenon, deep vein thrombosis (DVT), stroke, tardive dyskinesia, dyslexia, brain injury, and vertigo. Previously five polymorphic forms of piracetam have been reported, but two of these (Form IV (8.954) and Form V (6.390)) have only been obtained at high-pressure (>0.5 GPa) conditions.^15^ The remaining three polymorphs (Form I (6.747), Form II (6.403), and Form III (6.525)) have been identified and structurally characterized under ambient conditions.^16^ For clarity, the numbers within parentheses refer to the unit cell dimension along the A-axis in Å. The trimorphic thermodynamic stability relationship is rather complex, which despite being well-studied is not completely elucidated.^17^ Out of the three polymorphs, Form I is unstable below 383.15 K and can be isolated only by heating Form II or Form III to 400 K and then quenching to room temperature, which however typically leads to transformation back to Form II within a few hours. Form I is thus not of much practical relevance. At ambient temperature, Form II is metastable, and Form III is the stable polymorph. Form I and Form III are enantiotropically related, with a reported transition temperature at 393 K.^17^ The solubilities of Form II and Form III have been determined in a range of organic solvents^18,19^ together with an investigation into the polymorphic transformation behavior. These studies have shown that there is no transition in stability between these two polymorphs over the temperature range 278–323 K. Kuhnert-Brandstatter et al.^20^ have proposed that all three polymorphs are enantiotropically interrelated, with Form II and Form III having a transition temperature greater than 348.15 K, but because both forms tend to transform to FI at high temperatures, there is only indirect experimental evidence for the existence of such a transition temperature.^17^ In addition to the ansolvate forms, crystallization experiments in water have revealed the existence of a monohydrate form^15^ of piracetam, as well as a dihydrate form^21^ that has however only been obtained at high pressure.

Various analytical techniques like powder X-ray diffraction (PXRD), *in situ* energy dispersive X-ray diffraction, *in situ* Raman spectroscopy, infrared spectroscopy (IR), and near-infrared spectroscopy have been used to investigate the solid-state, solution-mediated, and wet granulation-induced polymorphic transformations of piracetam.^17,20,22−27^ Recently, our group published a detailed study on the crystal growth kinetics of the metastable Form II and the stable Form III in two organic solvents.^28^ However, to the best of our knowledge, no crystal nucleation induction time study of piracetam has been reported so far .

During preliminary nucleation experiments in ethanol and isopropanol, a new crystal polymorph (Form VI) was encountered. The new Form VI has been characterized using X-ray powder diffraction (PXRD), Raman spectroscopy, attenuated total reflectance infrared spectroscopy (ATR-IR), and differential scanning calorimetry (DSC). A total of 1200 isothermal nucleation induction time experiments have been performed under different conditions of temperature and concentration, resulting in Form VI nucleating in the majority of cases. The solubility of Form VI has been determined by a gravimetric method, and the nucleation parameters of the new polymorph were determined in ethanol and isopropanol.

## 2 Experimental Section

### 2.1 Materials

Piracetam (PCM Form III, 2-(2-oxopyrrolidin-1-yl)-acetamide, 99.9% wt., CAS number 7491-74-9) was supplied by Baoji Guokang Bio-Technology Co., Ltd., Baoji, China, complying with European Pharmacopoeia standards EP 6.0. The solvents used were ethanol (EtOH, 99.8% GC, CAS number: 64-17-5), and isopropanol (IPrOH, 99.98%GC, CAS number: 67-63-0), both supplied by Fisher Scientific Ltd. All chemicals were used as received without further purification.

### 2.2 Polymorph Characterization

PXRD, solid-state Raman spectroscopy, differential scanning calorimetry (DSC), and ATR-IR have been used to characterize Form III (as received), Form II, and Form VI (obtained in nucleation experiments). Crystals of the three polymorphs have also been characterized using optical microscopy (Olympus XI53) and scanning electron microscopy (JEOL JCM-5700). Transmission powder X-ray diffractograms were recorded using an Empyrean diffractometer (Malvern PANalytical Ltd..) with Cu Kα_1,2_ radiation (λ = 1.5406/1.5444 Å) operating at 40 kV and 40 mA at room temperature. Samples were scanned from 13° to 35° (2θ) with 0.0066° 2θ/min step size and 48.19 s per step, on a flat stage that was spinning at 4 rpm on transmission mode. Reflection X-ray powder diffractograms were recorded using an Empyrean diffractometer (Malvern PANalytical Ltd.) with Cu Kα_1,2_ radiation (λ = 1.5406 Å) operating at 40 kV and 40 mA at room temperature. Samples were scanned from 13° to 35° (2θ) with 0.026° 2θ/min step size and 112.97 s per step, on a flat stage that was spinning at 4 rpm on reflection mode. *Ex situ* Raman spectroscopy was performed using a probe Mettler Toledo spectrometer. For each spectrum, five scans were collected for 30 s each from 4000 to 100 cm^–1^ at 1 cm^–1^ resolution using iCRaman software v4.3. *Ex situ* infrared spectroscopy was performed using a PerkinElmer Spectrum One spectrophotometer with an ATR accessory equipped with a ZnSe sample plate. For each spectrum, four scans were collected from 4000 to 650 cm^–1^ at a resolution of 4 cm^–1^. DSC was performed using a Netzsch Polyma DSC 214 instrument, with Concavus Al pans with pierced lids. All runs were performed at a heating rate of 10 K min^–1^ heating rate, from 293 K to 443 K.

### 2.3 Induction Time Experiments

Initially, 240 induction time experiments (Set I) were carried out at different solute concentrations and temperatures in the range 283.15–298.15 K in ethanol only, with 40 repeat experiments at each condition. The concentration and temperature combinations were selected somewhat arbitrarily, with the primary goal to find a suitable basis for an investigation into the kinetics of nucleation of piracetam. A further 960 induction time experiments (Set II) were then carried out at 288.15 K in ethanol and isopropanol, with 80 repeat experiments at each condition, to estimate the interfacial energy, pre-exponential factor, and solubility of the new polymorph.

An agitation rate of 200 rpm was used in all the experiments. Temperature control and agitation were achieved using thermostatic water baths (Grant, GR150-S26 stirred with pump and a C2G cooling unit) equipped with submersible multipole magnetic stirrer plates. The experiments were carried out in batches of 40 simultaneous induction time experiments, in glass vials (VWR, 70.5 × 27.5 mm). Two water baths, each with a submersible 20-pole magnetic stirrer plate, were set at the nucleation temperature, and one water bath with a submersible 40-pole magnetic stirrer plate was used for dissolution. For the nucleation experiments, all the 40 vials were moved from the high-temperature water bath to the low-temperature water baths (20 vials in each bath). The vials were recorded using a high-definition camcorder (Sony HDRXR520VE). The visible onset of nucleation was characterized by sharp transition from a clear to a completely opaque solution within 5–10 s. The induction time was taken as the time interval between the insertion of a vial into the bath at the nucleation temperature and the first change noticed in the sample, as observed by the naked eye from the camcorder recordings. As soon as each specific vial had visibly nucleated, the suspension was filtered within ∼2 min using a filter paper (Whatman), and the solids were dried on the filter paper at room temperature for 24–72 h in a laboratory fume hood to complete dryness. The samples were then analyzed for the polymorphic form using PXRD and Raman spectroscopy, with the dried powder samples used directly with only light or no grinding to avoid polymorphic transformation. From some vials, only half of the sample was filtered, and the remaining 5 mL was analyzed after ∼15 min to investigate if polymorphic transformation had occurred.

A reference vial, stirred, containing only the solvent and an *in situ* temperature probe (Dostmann model number P600) was placed in the bath at the nucleation temperature along with the other vials, in order to determine the time for the solution in a vial to reach the nucleation temperature. A total of 260–315 s was required to reach the nucleation temperature to within ±0.5 K, and 385–425 s to reach the nucleation temperature to within ±0.1 K.

#### 2.3.1 Set I

Solutions of piracetam (starting with Form III) using selected concentrations were prepared in two glass bottles (sealed using plastic screw caps) with a 200 mL stock solution in each bottle. The stock solutions were agitated with PTFE-coated magnetic stir bars and equilibrated for 4 h at a temperature 5 K above the temperature corresponding to saturation with respect to Form III.^18^ From the respective stock solution, 10 mL aliquots were filtered into 40 vials using preheated syringes and 0.2 μm PTFE filters (Millipore), taking care not to induce nucleation during the preparation and maintaining solution temperatures above the respective saturation temperature. To each vial, a magnetic stir bar (Sigma-Aldrich, polygon-shaped, 1/2 × 1/8 in.) was added before sealing with a plastic screw cap with a PTFE seal. After sealing, the vials were equilibrated overnight (∼16 h) under agitation at 5 K above the Form III saturation temperature, before being transferred to the nucleation baths.

#### 2.3.2 Set II

For the second set of experiments (Set II), stock solutions of PCM were prepared in six different concentrations each in ethanol and isopropanol. From each of the stock solutions, 20 mL samples were filtered into 80 glass vials using the procedure and equipment specifications described in section 2.3.1. As for Set I, the capped vials were equilibrated overnight (∼16 h) under agitation at 5 K above the saturation temperature, before being transferred to the nucleation baths.

### 2.4 Solubility Measurement

An estimate of the solubility of Form VI in ethanol and isopropanol at 288.15 K was obtained using a previously described^29,30^ gravimetric procedure. In the present work, equilibrium was reached by nucleation and growth rather than by dissolution. Following nucleation, 2 mL solution samples were extracted from the vials at intervals of 2 min, starting from the point when the solutions turned cloudy. To facilitate solid–liquid separation, stirring was switched off, and liquid samples were collected from the clear solution at the top. Samples of the solid phase present were also collected and analyzed with PXRD to verify the polymorphic form. Within 8 min, the solution concentration stabilized, and up to this point there was no change in the PXRD pattern, indicating that no polymorph transformation had taken place. Transformation from Form VI to Form II was detected 15 min after nucleation in ethanol, as shown in Figure 3, while no transformation occurred in isopropanol for at least 6 h. The samples were dried for at least 3 days or until all solvent had evaporated, as verified by regular recording of the vial weights. Once dry, the final mass of each vial was recorded, and the solution concentration in mole fraction was calculated from the initial and final masses. Solubility values were also converted to mol L^–1^ using eq 1, assuming that the density of the solution can be approximated by the density of the pure solvent, 0.789 g L^–1^ for ethanol and 0.785 g L^–1^ for isopropanol. The final solubility estimates were taken as the average of three samples and are reported in Table 3.
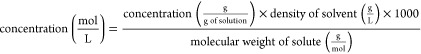
1

## 3 Results

### 3.1 Polymorph Characterization

**Figure 2:**
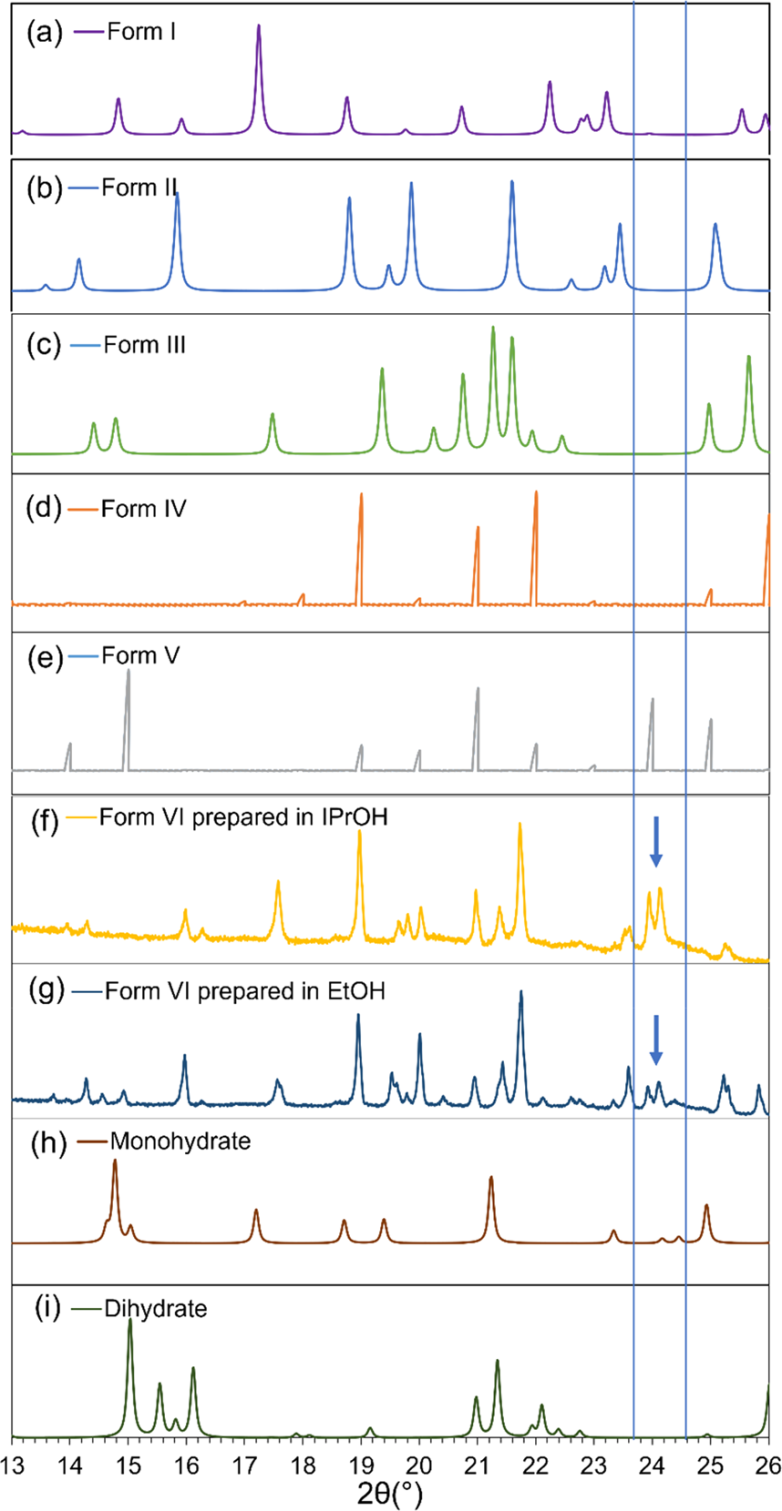
(a–i) PXRD diffractograms of dried Form VI obtained from isopropanol and ethanol using the method described in section 2, compared to PXRD patterns of other polymorphs obtained from structures in the CSD; Form I: BISMEV03, Form II: BISMEV, Form III: BISMEV01, Form IV: BISMEV04, Form V: BISMEV07, monohydrate: YAKWAJ, and dihydrate: LIFNOE. Arrows indicate the position of a peak distinguishing Form VI from the other known solid forms.

Figure 2 shows the transmission PXRD diffractograms of Form VI of piracetam obtained in the present work by nucleation from ethanol and isopropanol solutions at 298.15 K, together with the theoretical PXRD patterns of Forms I, II, III, IV, and V, and the reported monohydrate and dihydrate forms, obtained from structures deposited in the Cambridge Structural Database (CSD). As shown in Figure 2, a unique, characteristic peak at a 2θ-value of 24.2° was observed for the new polymorph. More reflection PXRD graphs obtained from nucleation experiments for this polymorphic form are shown in Figure S1 (Supporting Information).

**Figure 3:**
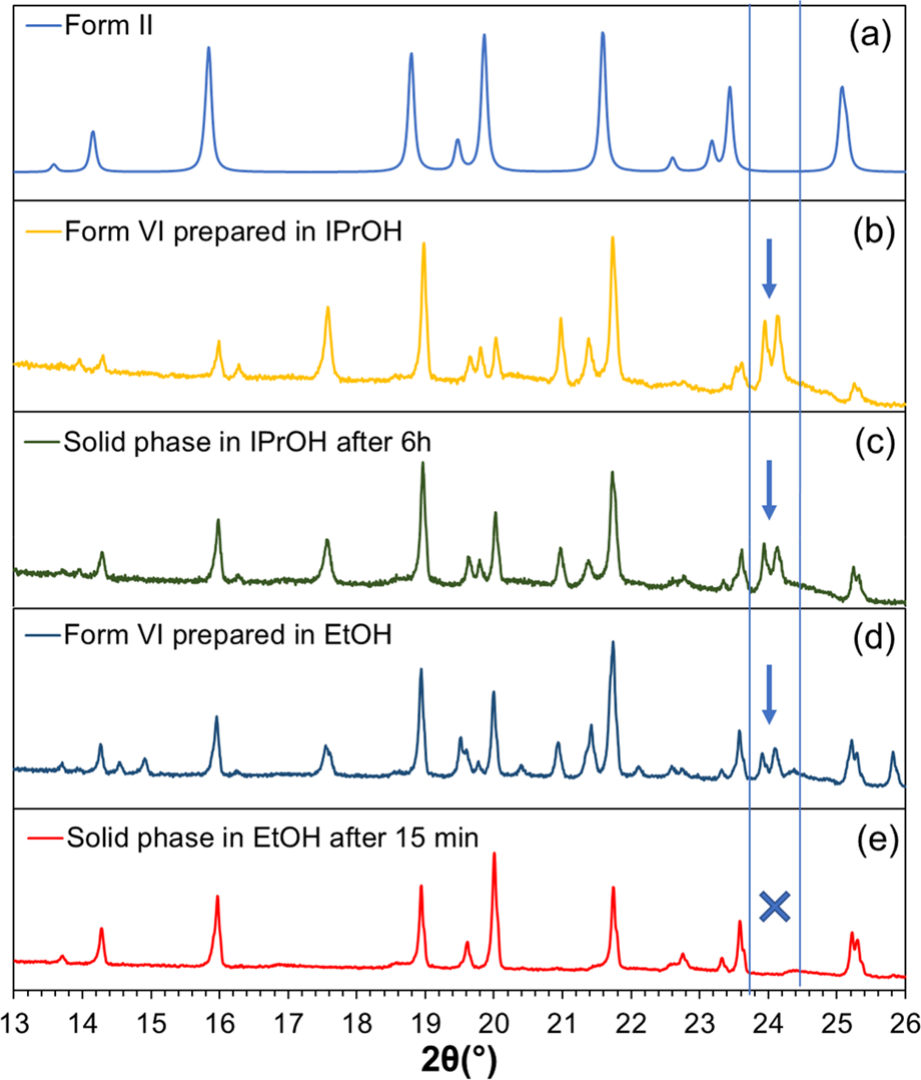
(a–e) Transmission PXRD diffractograms of dried Form VI obtained from isopropanol and ethanol immediately after nucleation, after 6 h for isopropanol and after 15 min in ethanol in stirred suspension before filtration, compared to PXRD patterns of other polymorphs obtained from structures in the CSD; Form II: BISMEV, Form III: BISMEV01 Arrows indicate the characteristic peaks of Form VI.

Figure 3 shows PXRD diffractograms of solids nucleating as Form VI sampled immediately after nucleation and following additional time in an agitated suspension, compared with diffractograms of Form II and Form III obtained using crystal structures available in the CSD. In ethanol, Form VI is verified to have completely transformed into Form II after 15 min, but in isopropanol Form VI remains unchanged for at least 6 h of observation.

**Figure 4:**
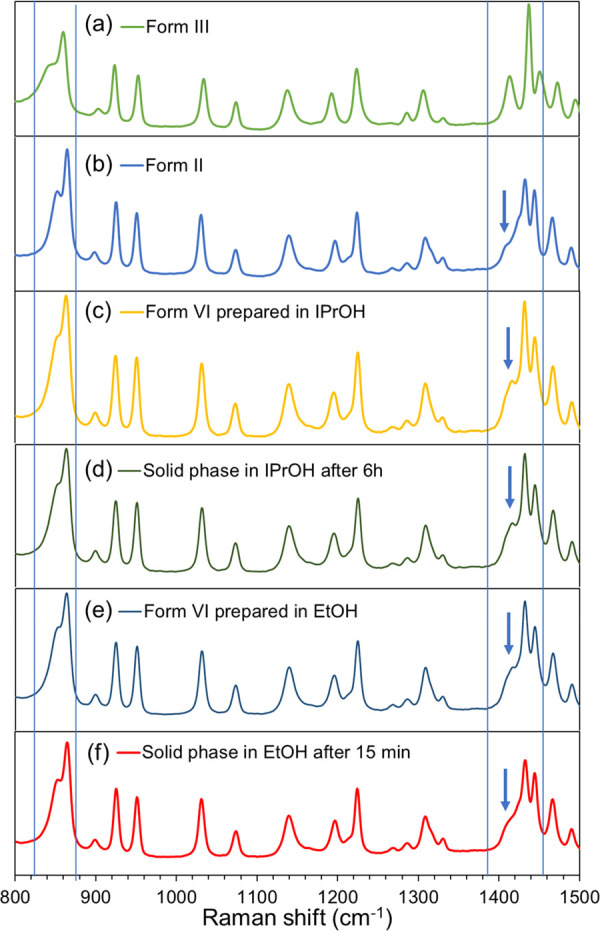
Solid-state Raman spectra of (a) Form III as received, (b) Form II obtained in this work, and Form VI obtained immediately after nucleation in (c) isopropanol and (e) ethanol and following a further 6 h and 15 min in stirred suspension in (d) isopropanol and (f) ethanol, respectively. Arrows indicate the location of the characteristic Raman peak value distinguishing Form VI from Form II.

Figure 4 compares Raman spectra of different piracetam polymorphs. The spectra obtained for Form III and Form II match perfectly with spectra already reported for these forms.^31^ Characteristic Raman shifts of Form III are observed at 843 cm^–1^, 862 cm^–1^, 1416 cm^–1^, and 1438 cm^–1^, and of Form II at 853 cm^–1^, 866 cm^–1^, 1424 cm^–1^, 1408 cm^–1^, and 1434 cm^–1^. Raman shifts of Form VI are observed at 851 cm^–1^, 865 cm^–1^, 1418 cm^–1^, and 1433 cm^–1^, differing from the other polymorphs mainly with respect to the peak at 1418 cm^–1^ (representing C–H symmetric bending) which is found at 1408 cm^–1^ for Form II and at 1438 cm^–1^ for Form III. The Raman spectrum of Form VI is more similar to that of Form II than that of Form III. The results obtained with Raman spectroscopy agree with PXRD in that there is no change in the solid form obtained from isopropanol solution immediately after nucleation and after 15 min in suspension, as shown in Figure 4d. In ethanol suspension, the Raman spectrum obtained after 15 min indicates a complete transformation into Form II, as shown in Figure 4f. Additional solid-state Raman spectra of Form VI obtained from nucleation are shown in Figure S2 (Supporting Information). The full Raman spectra of all the forms presented here are shown in Figure S3 (Supporting Information).

**Figure 5:**
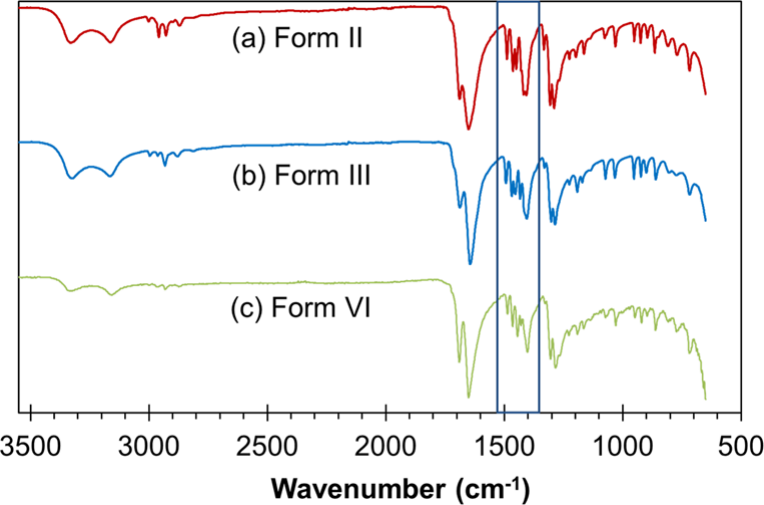
ATR FTIR spectra of (a) Form II obtained in this work, (b) Form III as received, and (c) Form VI obtained in this work. The region where differences could be expected to be found between the three polymorphs is highlighted.

Figure 5 shows the ATR infrared spectra of crystals of Form II, Form III, and Form VI. As for Raman spectroscopy, the three spectra exhibit very small differences. The main region of distinguishing features between these three polymorphs is located in the range 1400–1500 cm^–1^.

DSC thermograms obtained for the three solid phases (Form II, Form III, and Form VI; shown in Figure S4 of the Supporting Information) exhibit qualitatively similar behavior; a weak endothermic transformation peak with an onset between 390 and 400 K, followed by a melting peak with an onset at around 426 K, corresponding to the reported melting point of Form I.^20,27^

The estimated solubility of Form VI in ethanol and isopropanol at 288.15 K is summarized in Table 3. In mole fraction terms, the estimated solubility is higher in ethanol than in isopropanol, in agreement with data reported for Form II^19^ and Form III.^18^ At 288.15 K, the solubility ratios in terms of mole fraction solubility of Form VI: Form II: Form III is 1.195:1.080:1.000 ± 0.004 in ethanol and 1.196:1.090:1.000 ± 0.006 in isopropanol. It should be stressed here that the relative uncertainty in the Form VI solubility values is higher compared to the Form II and Form III data because of the difference in method (approaching equilibrium by nucleation and growth) as well as the short equilibration time (necessary to avoid transformation).

**Figure 6:**
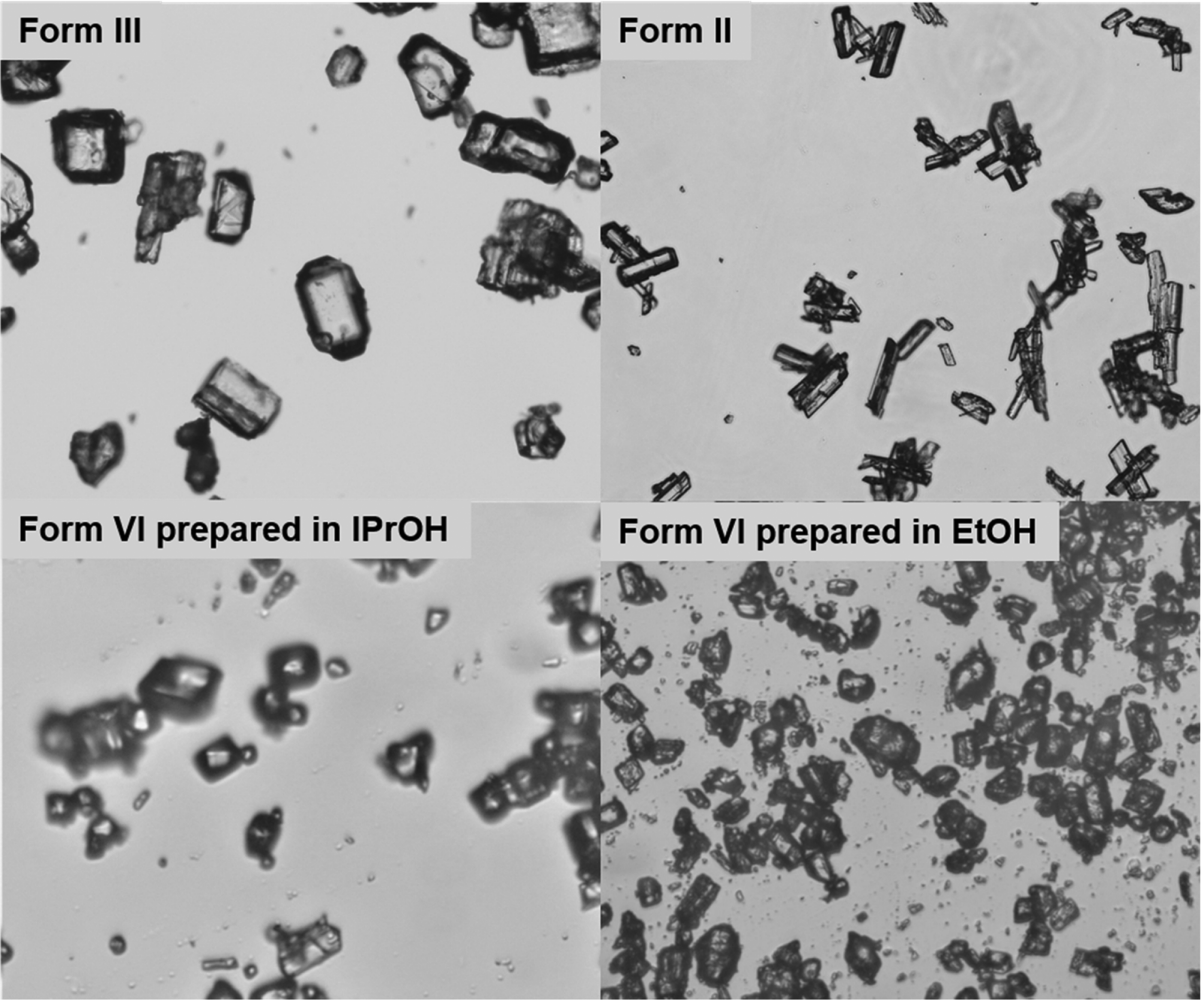
Optical micrographs of Form III (as received), Form II, and Form VI obtained from isopropanol and ethanol.

**Figure 7:**
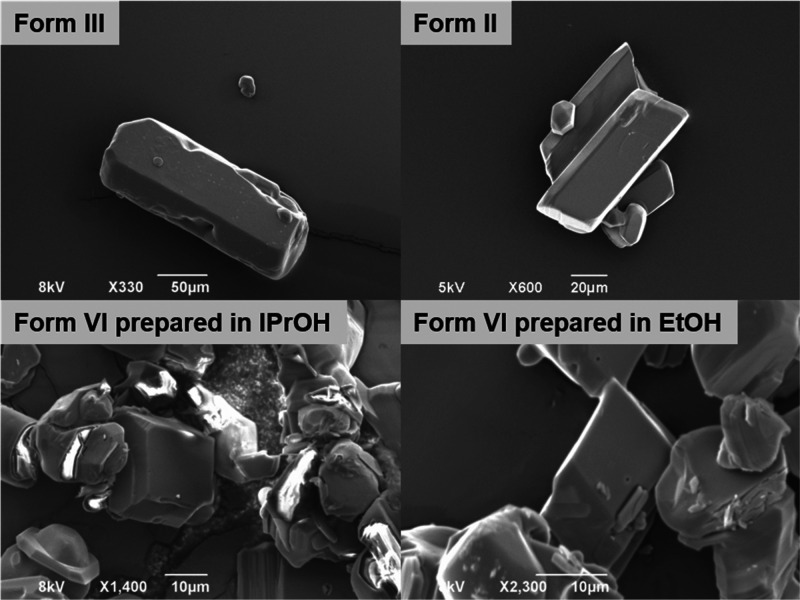
SEM images of Form III (as received), Form II, and Form VI obtained from isopropanol and ethanol.

Four samples of the different polymorphs of piracetam were analyzed further using optical microscopy and scanning electron microscopy, shown in Figure 6 and Figure 7. The samples of Form II were obtained by crystallization in ethanol and Form VI in ethanol and isopropanol, while Form III was used as received. The figures show that the habit of Form III and Form VI (more clearly seen in samples from isopropanol) is relatively blocky, while the habit of Form II is more plate-like, for crystals from both solvents.

### 3.2 Nucleation

**Figure 8:**
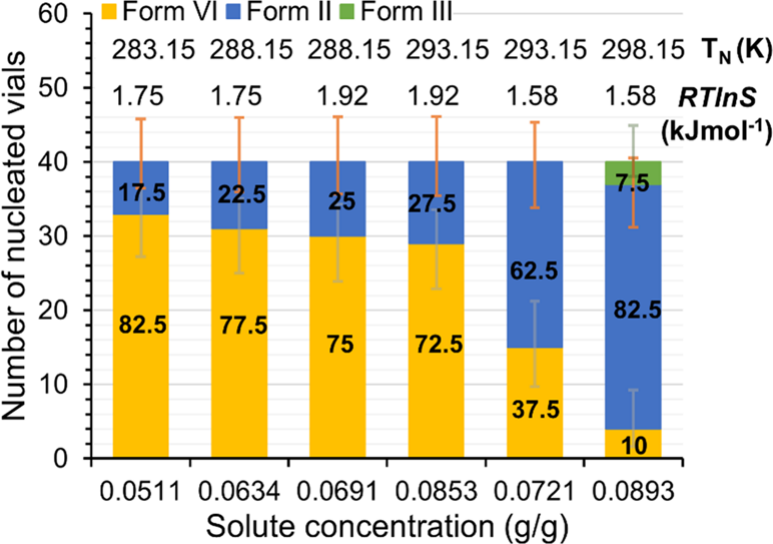
Polymorphic outcome of Set I experiments in ethanol at different nucleation temperatures and solute concentrations, along with the nucleation temperature (*T*_N_) and nucleation driving force with respect to Form VI. The fraction of vials (%) nucleated as each respective polymorph are given as numerical values, and error bars show 95% confidence limits calculated using the Wilson method^32^ with no correction for continuity.

Figure 8 shows the fractions of Set I experiments resulting in each polymorphic form for ethanol solutions nucleating at different temperatures and concentrations. In all the Set I experiments, all 40 vials nucleated within 2.5 h. Moreover, each vial resulted in a single polymorphic form, i.e., the material in each vial was polymorphically pure, but different vials were observed to yield different polymorphs. Samples from each vial were collected by filtering within 5 min of the cloud point being attained in the vial to ensure no further transformation. The results clearly show changes in experimental conditions affecting the polymorphic outcome in statistical terms. As the sample size was relatively small, and as concentrations and temperature combinations were selected somewhat arbitrarily primarily to find a suitable basis for the Set II experiments, it is not straightforward to draw clear conclusions about the influence of individual process conditions. In the figure, the driving force is given with respect to Form VI, for 288 K as obtained using the experimentally determined Form VI solubility, and for other temperatures as calculated from the solubility of Form III and using the solubility ratio Form VI/Form III at 288.15 K. The driving force is expressed as a chemical potential difference, Δμ, estimated using eq 2 by approximating activities with concentrations:
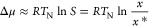
2where *T*_N_ is the nucleation temperature, and *S* is the supersaturation ratio approximated as the ratio of mole fraction concentrations in the supersaturated solution (*x*) and at saturation (x*). The corresponding driving forces with respect to Form II and Form III for each experiment can be calculated from the solubility ratios given above.

**Table 1:** Polymorphic Outcome of Eighty Vials Nucleated in Set II Experiments Carried out at Different Driving Forces for Nucleation (with Respect to Form VI):

ethanol						
Δμ (kJ mol^**–**1^)	1.40	1.46	1.51	1.61	1.67	1.72
Form VI	69 (86.2%)	68 (85.0%)	68 (85.0%)	67 (83.8%)	66 (82.5%)	66 (82.5%)
Form II	11 (13.8%)	12 (15.0%)	12 (15.0%)	13 (16.2%)	12 (15.0%)	10 (12.5%)
Form III					2 (2.5%)	4 (5.0%)

isopropanol						
Δμ (kJ mol^**–**1^)	1.50	1.67	1.73	1.84	1.96	2.07
Form VI	75 (93.8%)	74 (92.5%)	74 (92.5%)	72 (90.0%)	71 (88.8%)	70 (87.5%)
Form II	5 (6.2%)	6 (7.5%)	6 (7.5%)	8 (10.0%)	9 (11.2%)	10 (12.5%)

In order to evaluate the nucleation kinetics of Form VI, a low nucleation temperature (288.15 K) was chosen for a second set (Set II) of induction time experiments in ethanol and isopropanol solutions. In Set II, all 80 vials nucleated within the experimental time frame, and each set of repeat experiments resulted in proportions of vials each nucleating as a single polymorph. The polymorphic outcome is given in Table 1. All conditions resulted in the majority of vials nucleating as Form VI: > 83% of the vials in ethanol and >88% in isopropanol.

**Figure 9:**
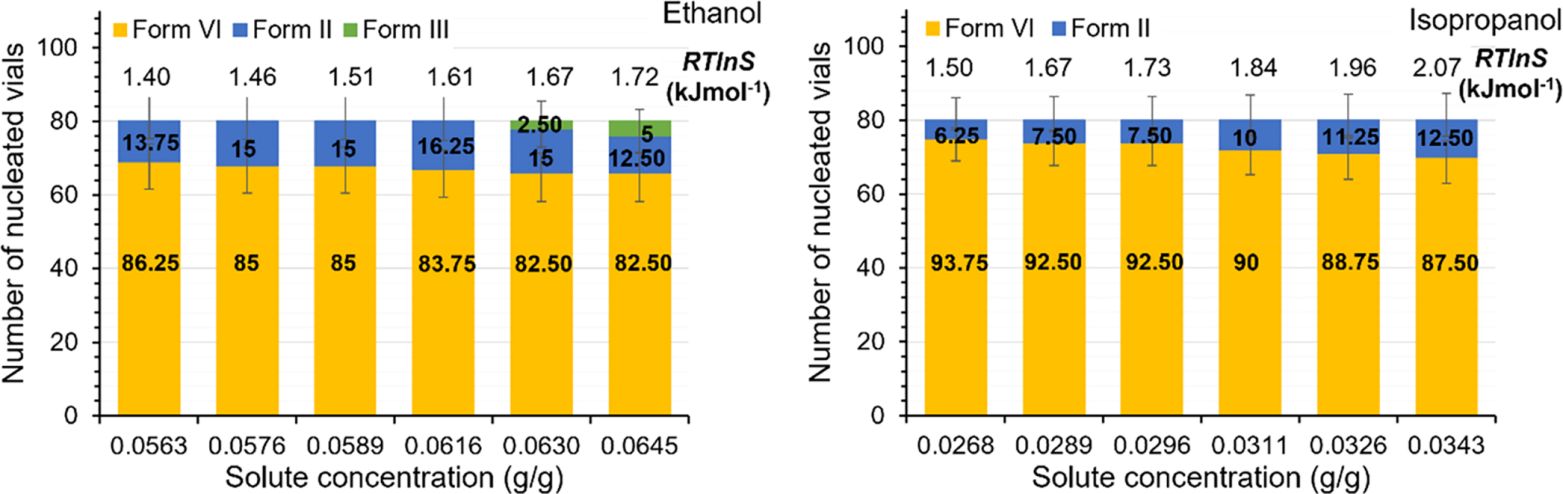
Polymorphic outcomes at different solute concentrations and nucleation driving forces, at 288.15 K in (left) ethanol and (right) isopropanol (Set II). The fraction of vials (%) nucleated as each respective polymorph are given as numerical values, and error bars show 95% confidence limits calculated using the Wilson method^32^ with no correction for continuity.

Figure 9 shows the fractions of Set II experiments resulting in each polymorph for different supersaturation values. The supersaturation is quantified as the thermodynamic driving force for nucleation of Form VI, given by eq 2, and using the experimentally determined mole fraction solubility (x* in Table 3) of Form VI at 288.15 K. In both solvents, there is a weak but consistent trend showing a larger proportion of vials nucleating as Form VI with decreasing driving force. In isopropanol, the fraction of vials nucleating as Form II correspondingly increases with increasing driving force, while in ethanol the proportion of Form II remains less affected. In ethanol, some vials nucleate as the stable Form III at higher driving forces, but this polymorph was not encountered at all in isopropanol. Both these trends are against expectation, i.e., that the formation of a more unstable form would be promoted by a higher supersaturation. However, it should be stressed that these trends cannot be confidently established within the limits of experimental uncertainty.

**Figure 10:**
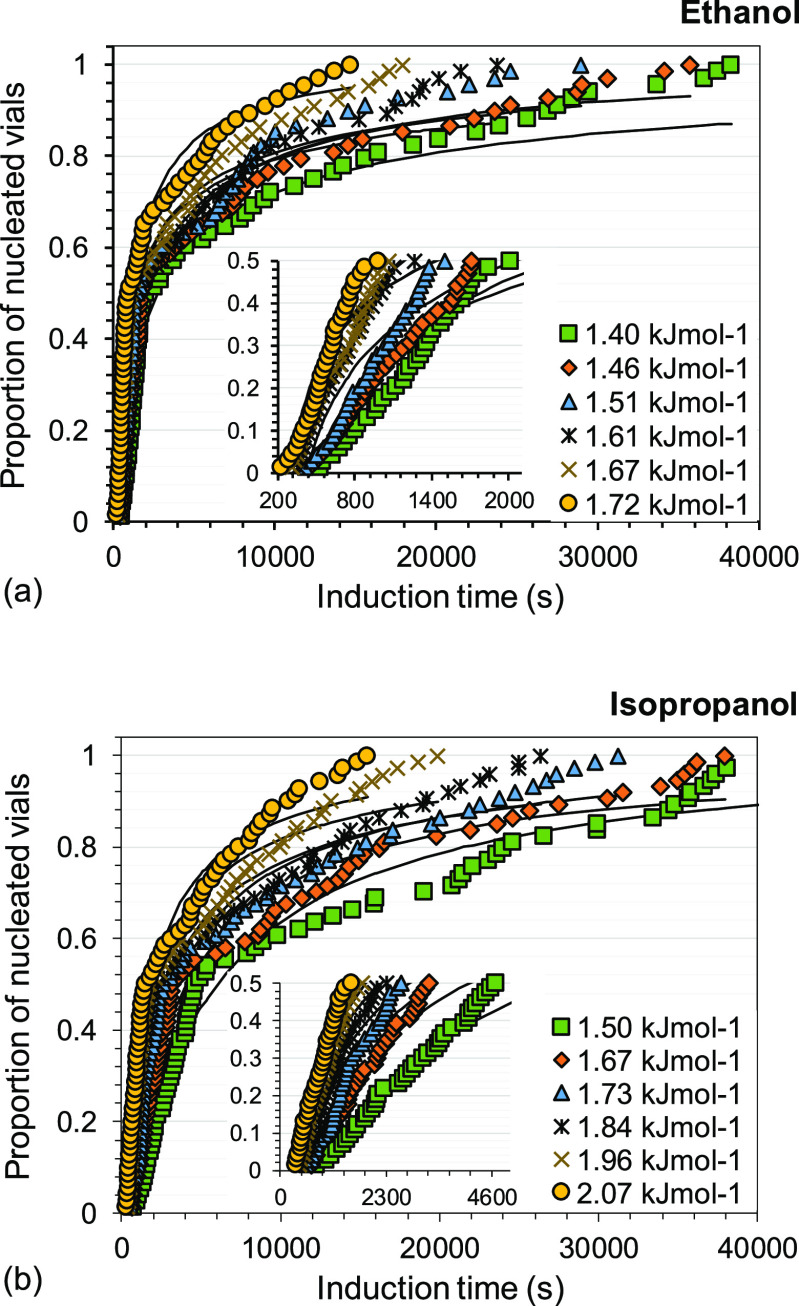
Induction time distributions of Form VI in (a) ethanol and (b) isopropanol at a nucleation temperature of 288.15 K at different driving forces with fitted log-normal cumulative distribution functions (black solid lines). Each data point represents the induction time obtained in one single experiment.

The induction time distributions for the experiments nucleating as Form VI in ethanol and isopropanol at 288.15 K are shown in Figure 10. Log-normal cumulative distribution functions are fitted to the experimental data, with overall good fits (*R*^2^ > 0.97) as seen in the figure.

**Figure 11:**
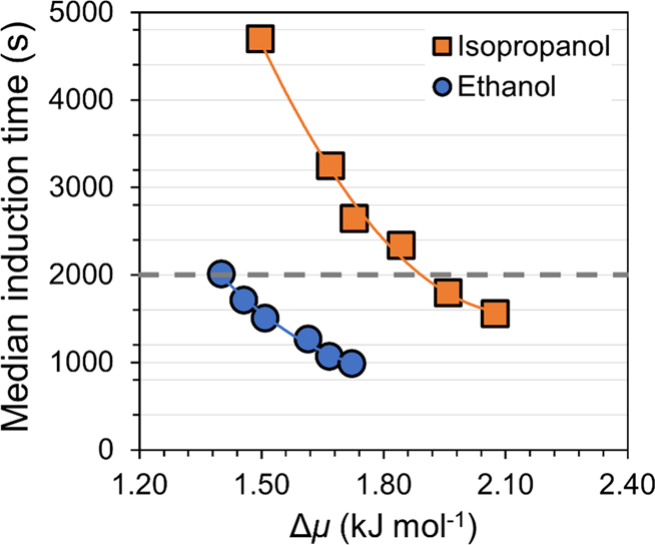
Influence of the nucleation driving force on the median induction time for Form VI in ethanol (blue, ●) and isopropanol (orange, ■), from Set II experiments, together with exponential trend lines.

The median induction time, extracted directly from the experimental induction time distributions, for nucleation of Form VI in each experiment was used as input for further analysis of nucleation parameters. Figure 11 shows a plot of experimentally obtained median induction times against the driving force for nucleation of Form VI in ethanol and isopropanol. The median induction time values are summarized in Table 2. Median induction time values are calculated using mole fraction solubilities (x*) mentioned in Table 3.

**Table 2:** Median Induction Times of Form VI Obtained at Different Driving Forces, at a Nucleation Temperature of 288.15 K (Set II)

ethanol						
Δμ (kJ mol^**–**1^)	1.40	1.46	1.51	1.61	1.67	1.72
induction time (s)	2013	1711	1506	1269	1071	986

isopropanol						
Δμ (kJ mol^**–**1^)	1.50	1.67	1.73	1.84	1.96	2.07
induction time (s)	4698	3246	2645	2337	1794	1555

**Table 3:** Solid–Liquid Interfacial Energy (γ), Pre-exponential Factor (*A*), Solubility at 288.15 K (g g^–1^, mol L^–1^ (C*) and Mole Fraction (x*)) of Form VI, Solvent Normal Boiling Points, and Pre-Exponential Factor (*A*/*A*_1_) Calculated Using eq 6 from Physical Properties in Ethanol and Isopropanol

solvent	***γ*** (mJ·m^**–**2^)	*A***(**m^–3^·s^–1^)	C* (g g^**-1**^, mol L^**–1**^) at 288.15 K	x* at 288.15 K	solvent normal boiling point (K)	*A*/*A*_1_ ((g·mol^**–**1^)^0.5^·(mol·L^**–**1^)·(mPa·s)^**–**1^·(mJ·m^**–**2^)^−0.5^)
ethanol	4.15	75	0.0311	0.00998 ± 0.00006	351.52	0.623
			0.173			
isopropanol	4.45	35	0.0143	0.00601 ± 0.00003	355.65	0.148
			0.079			

As shown in Figure 11, in terms of the thermodynamic driving force required, the barrier to Form VI nucleation is significantly lower in ethanol than in isopropanol. The estimated driving force required to reach an induction time of 2000 s (indicated by the dashed line in the figure) is 1.48 kJ mol^–1^ in ethanol and 1.89 kJ mol^–1^ in isopropanol. Thus, for this polymorph, the apparent nucleation difficulty increases in the order ethanol < isopropanol. However, at the same time, the *proportion* of Form VI obtained also increases in the order ethanol < isopropanol. This indicates that nucleation of Form II and Form III is even more obstructed in isopropanol compared to ethanol. In other words, the influence of the solvent on nucleation appears to be more pronounced for Form II and Form III than for Form VI.

**Figure 12:**
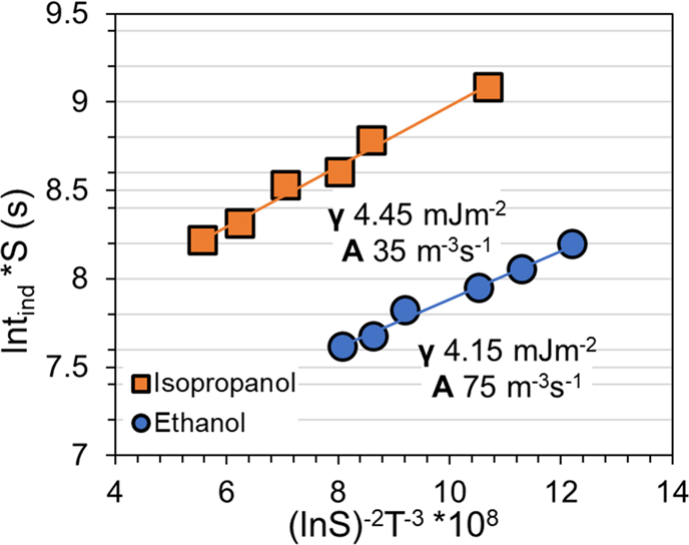
Classical nucleation plot of Form VI in ethanol (blue, ●) and isopropanol (orange, ■), together with respective linear correlations.

According to the classical nucleation theory (CNT),^33^ the rate of nucleation (*J*) as a function of *S* can be expressed as
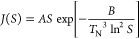
3where *T*_N_ (K) is the nucleation temperature, *A* (m^–3^s^–1^) is the pre-exponential factor, and *S* is the supersaturation ratio. Assuming the induction time to be equal to 1/*JV*:
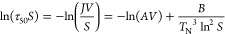
4where τ_50_ denotes the median induction time, extracted directly from the experimental induction time distributions, and *V* is the solution volume (20 mL). Figure 12 shows classical nucleation plots of ln(τ_50_*S*) against ln^–2^*S·T*_N_^–3^ for nucleation in both solvents. The pre-exponential factor (*A*) value is calculated from the intercept, and the interfacial energy (γ) is further calculated from the slope *B* through the equation:
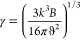
5where *k* (J·K^–1^) is the Boltzmann constant, and ϑ (m^3^) is the molecular volume. For all the calculations, the molecular volume of piracetam was taken as that in the crystal structure of Form II (CSD refcode BISMEV) equal to 1.74 × 10^–28^ m^3^. The values of the pre-exponential factor and the interfacial energy are given in Figure 12. The easier nucleation in ethanol is reflected in both a lower interfacial energy and a higher pre-exponential factor.

## 4 Discussion

As the comparison of PXRD patterns shows (Figure 2), Form VI is a new polymorphic form of piracetam. The structural changes that occur over time clarify that this polymorphic form is less stable than both Form II and Form III under ambient conditions. The stability relationship is also evident from a comparison of the solubility of the three forms in the same solvents. At 288 K, the estimated solubility of Form VI (0.0311 g g^–1^ in EtOH and 0.0143 g g^–1^ in IPrOH) as shown in Table 3 (C*) is higher than that of Form II^19^ (0.0287 g g^–1^ in EtOH and 0.131 g g^–1^ in IPrOH) and of Form III^18^ (0.026 g g^–1^ in ethanol and 0.012 g g^–1^ in isopropanol). The transformation of Form VI is slower in isopropanol than in ethanol, in line with the fact that the solubility in isopropanol is lower.

All these three polymorphic forms of piracetam (Form VI, Form II, and Form III) have been obtained in induction time experiments at different nucleation temperatures and solute concentrations. It can be concluded that Form VI is the overall most common result in the range of conditions evaluated herein. With increased nucleation temperature and associated higher concentrations, the proportion of experiments resulting in the more stable polymorphs (Form II and Form III) increases. This is in general agreement with observations reported for other systems,^13,34^ that with higher concentrations the greater the tendency to nucleate more stable forms, as a result of an interplay of thermodynamic and kinetic factors.

The driving force required to reach the same induction time increases in the order of solvents as ethanol < isopropanol. The estimated interfacial energy is lower, and the pre-exponential factor is higher for Form VI in ethanol compared to in isopropanol, as summarized in Table 3, with both parameters capturing that nucleation is easier in ethanol. Thus, from both the above statements, the ease of nucleation of this polymorphic form of piracetam increases in the order of solvents as isopropanol < ethanol. Moreover, given the fact that the proportion of Form VI obtained at equal driving force is higher in isopropanol than in ethanol, nucleation of Form II and Form III appears even more difficult in isopropanol.

A similar order between these two particular solvents has also been observed for nucleation of *n*-butyl paraben^35^ and fenoxycarb^36^ where the interfacial energy was found to be lower in ethanol compared to a propanol isomer (*n*-butyl paraben: 1-propanol and fenoxycarb: isopropanol). The difficulty of nucleation in isopropanol can be attributed to the fact that there is a stronger interaction between the solute and solvent molecules compared to ethanol, as shown in previous work by Maher et al.^17^ using molecular simulation.

In previous works^37^ on nucleation of inorganic compounds in water, an inverse relationship between the interfacial energy and the natural logarithm of the solubility was identified. This relationship is also found in the present work for the two evaluated solvents. However, in several other studies of a solute nucleating in different solvents, this relationship is not found.^38−40^ As shown in Table 3, the normal boiling point is higher for isopropanol compared to ethanol, similar to the order of interfacial energies of Form VI in the same solvents. A similar relationship has been observed in other studies,^35,41^ while there are also examples of the opposite.^36,38^

In previous work by our group^28^ for piracetam Form II and Form III in the same solvents, the interfacial energy calculated from growth experiments was found to be lower in ethanol (1.12 mJ m^–2^ Form II; 1.75 mJ m^–2^ Form III) and higher in isopropanol (1.18 mJ m^–2^ for Form II; 2.08 mJ m^–2^ for Form III), the same order between the solvents as obtained in the present nucleation work for Form VI (4.15 mJ m^–2^ in ethanol, 4.45 mJ m^–2^ in isopropanol). Interfacial energies are expected to increase with increasing thermodynamic stability of the polymorphic form. Accordingly, please note that the values for Form II and Form III are obtained from growth experiments, while the values for Form VI are from nucleation experiments. Values obtained from growth experiments are expected to be lower than those determined in nucleation experiments since in the former type the determination is related to a 2D nucleation on a crystal face, while the latter is related to a 3D nucleation in a solution.^42,43^

The pre-exponential factor is dependent on the attachment factor, the Zeldovich factor, and the concentration of nucleation sites.^40^ The attachment factor and the Zeldovich factor both depend on the interfacial energy. The attachment factor describes the rate by which molecules attach to the nucleus surface and depends on the rate of transport to the nucleus and the surface area of the nucleus. The surface area of the nucleus is governed by the interfacial energy and the supersaturation. The Zeldovich factor (*z*) is related to the interfacial energy (γ^–3/2^). Thus, the pre-exponential factor^33^ is dependent on interfacial energy as per eq 6:
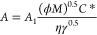
6where *M* (g·mol^–1^) is the solvent molecular weight, C* (mol·L^–1^) is the solubility obtained in this work as described in section 2.3.2, and *A*_1_ is a proportionality constant assumed to be independent of the solvent. η (mPa·s) is the solution viscosity at the nucleation temperature (288.15 K), here approximated by the solvent viscosity, and *ϕ* is the empirical parameter used for calculating diffusion coefficient using the Wilke–Chang equation, whose value is 1 for most solvents but 1.5 for ethanol.^44^ The obtained values of *A*/*A*_1_ are summarized in Table 3. Both this ratio as well as the experimentally determined pre-exponential factor values (*A*) exhibit a higher value in ethanol compared to isopropanol and thus with respect to the influence of the solvent show a correlation. Equation 6 indicates that with increasing interfacial energy the pre-exponential factor would of course be lower, and everything else unchanged. In the present work, a lower interfacial energy and a higher pre-exponential factor are found for ethanol solutions of Form VI compared to isopropanol solutions. Considering the fact that also the viscosity of isopropanol is higher, it is not surprising that the pre-exponential factor is lower in this solvent.

## 5 Conclusions

A new metastable polymorph (Form VI) of piracetam has been identified and characterized using PXRD, solid-state Raman, and IR spectroscopy. A characteristic peak in PXRD was identified at 24.2° distinguishing it from other known polymorphs. The new form nucleates preferentially over other polymorphs in both ethanol and isopropanol under the conditions investigated, but in ethanol Form VI crystals transform to Form II within approximately 15 min at 288 K, while in isopropanol the nucleated Form VI crystals remain unchanged for at least 6 h. At constant supersaturation, the proportion of experiments resulting in nucleation of Form VI shows an increasing trend with decreasing temperature of nucleation. Nucleation of Form VI in ethanol requires a lower thermodynamic driving force than in isopropanol to reach the same median induction time, the interfacial energy is lower, and the pre-exponential factor is higher in ethanol. However, the proportion of experiments nucleating as Form VI is higher in isopropanol than in ethanol.

## Supporting Information Available

Additional characterization data (PXRD, Raman, DSC) (PDF)

## Notations

*A*: pre-exponential factor
*A*_1_: arbitrary constant of pre-exponential factor
*B*: thermodynamic factor of nucleation
*C*: solute concentration
*C**: solubility
*J*: nucleation rate
*M*: molecular mass
*R*: gas constant
*S*: supersaturation
*T*_N_: nucleation temperature
*V*: volume
*x*: solute concentration (in mole fraction)
*z*: Zeldovich factor

## Greek letters

*τ*_50_: induction time
*γ*: interfacial energy
*η*: solution viscosity
*ϑ*: molecular/molar volume
