# Supplementary material for: Characterization and Crystal Nucleation Kinetics of a New Metastable Polymorph of Piracetam in Alcoholic Solvents
Source: Cryst Growth Des. 2022 Apr 22;22(5):2964–73. doi: 10.1021/acs.cgd.1c01421 (PMC9073936; doi:10.1021/acs.cgd.1c01421)
Supplement: Supplementary file 1 — cg1c01421_si_001.pdf [file cg1c01421_si_001.pdf]

Supporting information

Characterization and crystal nucleation kinetics of a new metastable polymorph of piracetam in alcoholic solvents

Shubhangi Kakkar[1]*, Lai Zeng*[2]*, Michael Svärd*[*,2]*, Åke C. Rasmuson*[1,2]

[1]*SSPC, Bernal Institute, Department of Chemical Sciences, University of Limerick, Limerick, Ireland.*

[2]*Department of Chemical Engineering, KTH Royal Institute of Technology, Stockholm, Sweden.*

* *Email: micsva@kth.se*

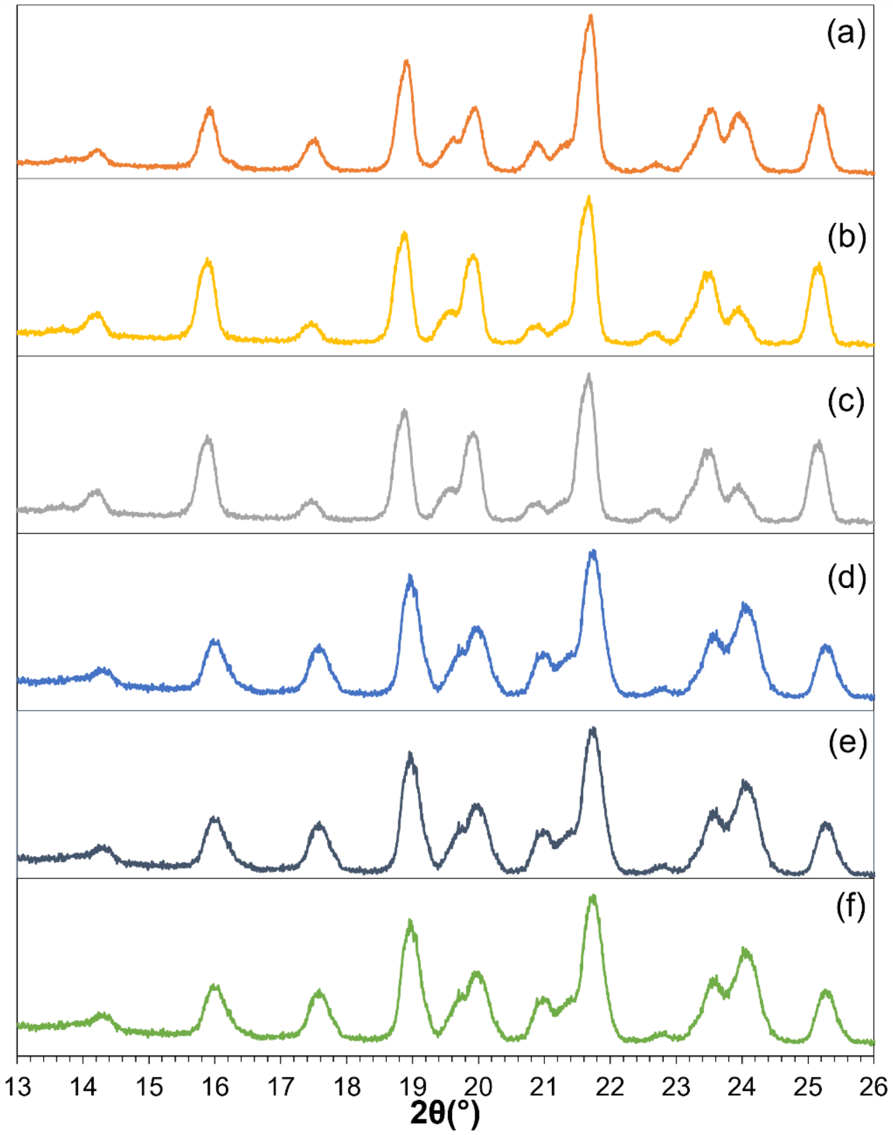

Figure S1. Additional reflection PXRD diffractograms of dried Form VI obtained from nucleation a) and b) Set I in ethanol, c) and d) Set II in ethanol and e) and f) Set II in isopropanol.

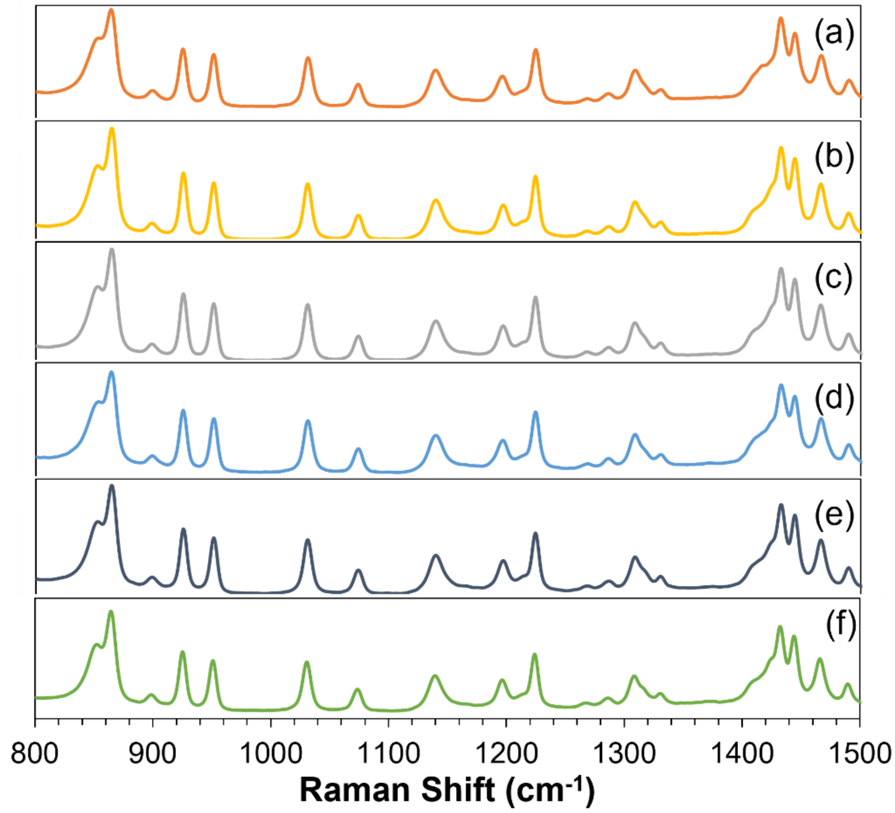

Figure S2. Additional solid-state Raman spectra of Form VI obtained from nucleation a) and b) Set I in ethanol, c) and d) Set II in ethanol and e) and f) Set II in isopropanol.

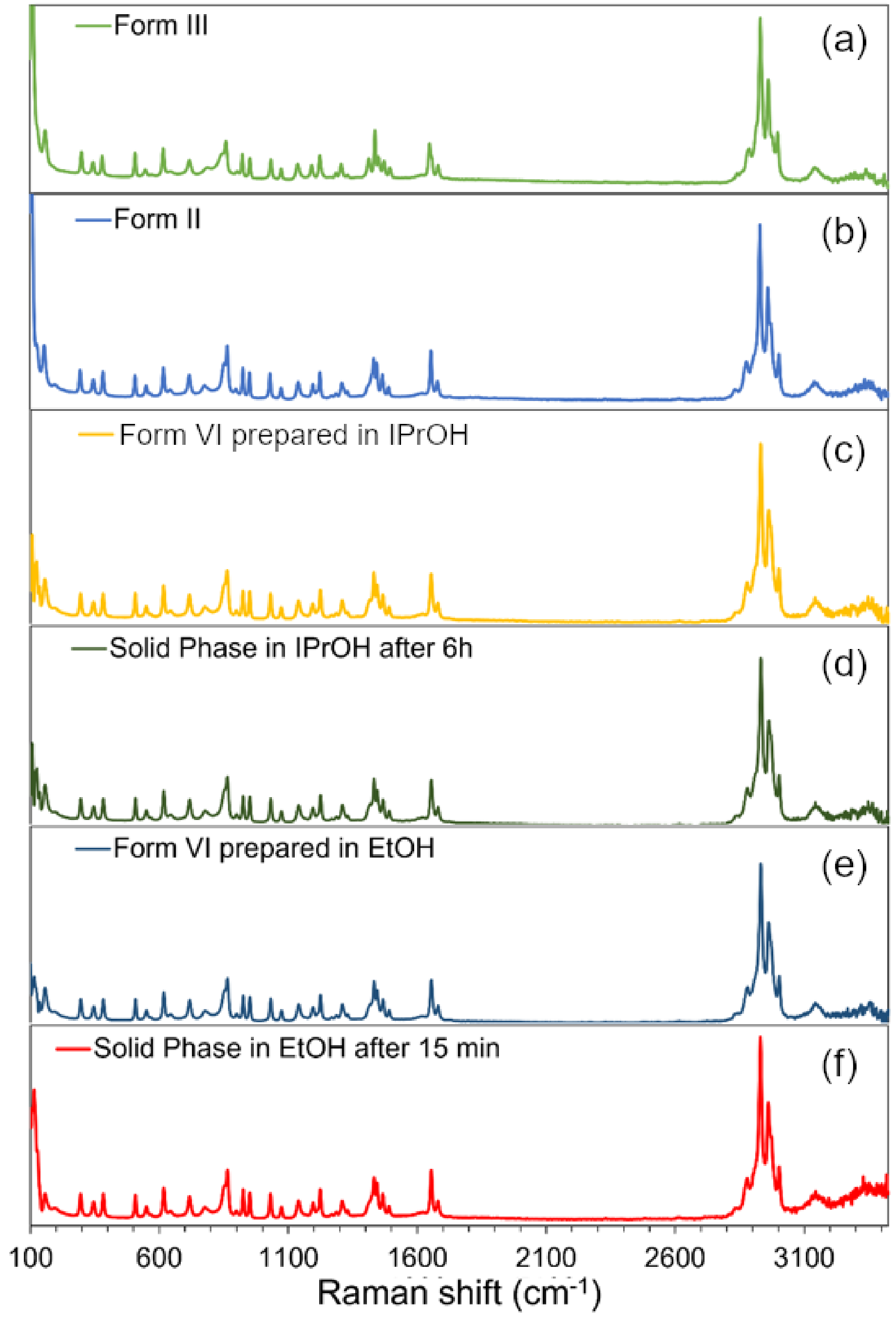

Figure S3. Full solid-state Raman spectra of a) Form III as received, b) Form II obtained in this work, and Form VI obtained immediately after nucleation in c) isopropanol and e) ethanol and following a further 6 h and 15 min in stirred suspension in d) isopropanol and f) ethanol, respectively.

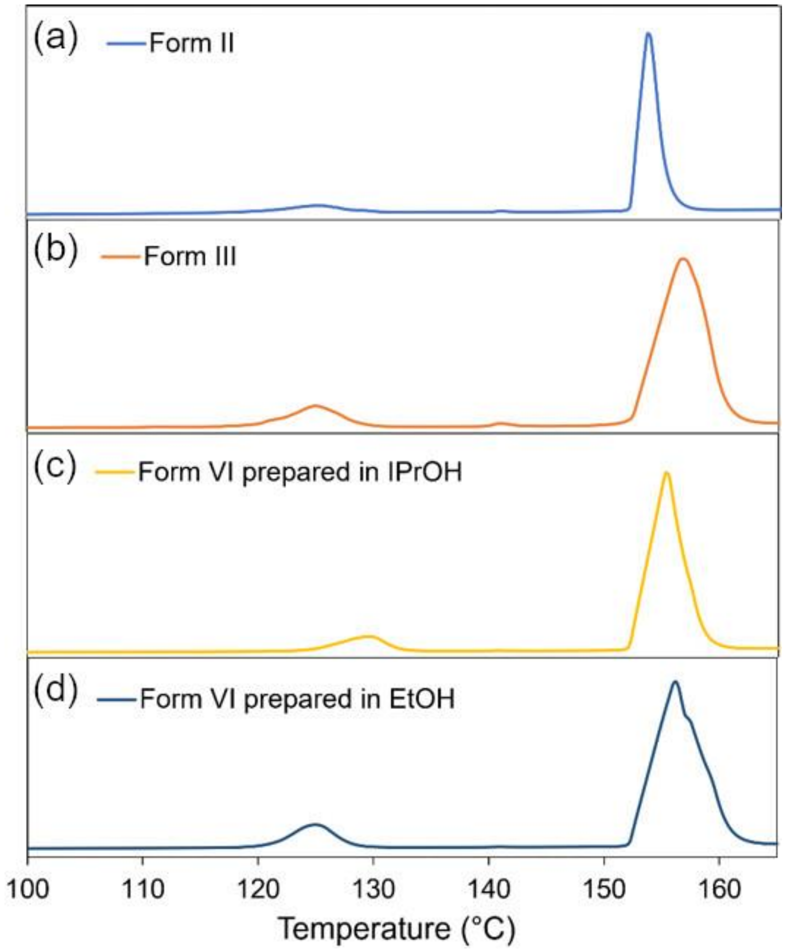

Figure S4. Standard DSC curves of a) Form II and b) Form III compared to Form VI prepared in c) isopropanol and d) ethanol. All runs were obtained at a heating rate of 10°C min^{-1}, from 20°C to 170°C.
